# Supplementary material for: Effects of anesthetics pentobarbital sodium and chloral hydrate on urine proteome
Source: PeerJ. 2015 Mar 12;3:e813. doi: 10.7717/peerj.813 (PMC4362303; doi:10.7717/peerj.813)
Supplement: Table S1 [file peerj-03-813-s001.docx]

**Supplemental Table 1. Urinary creatinine concentrations of all samples.**

| Pen pentobarbital sodium group | N (mmol/L) | A (mmol/L) |
| --- | --- | --- |
| R1 | 1.19 | 1.15 |
| R2 | 1.59 | 1.30 |
| R3 | 2.00 | 1.56 |
| R4 | 1.04 | 0.77 |
| R5 | 2.32 | 2.18 |
| R6 | 1.13 | 0.96 |
| Chloral hydrate group |  |  |
| R7 | 2.14 | 2.10 |
| R8 | 1.93 | 1.64 |
| R9 | 2.00 | 1.77 |
| R10 | 1.29 | 1.14 |
| R11 | 1.31 | 1.21 |
| R12 | 1.62 | 1.32 |

N: Normal condition; A: With anesthesia
